# Genome-wide identification of SNPs associated with body weight in yak

**DOI:** 10.1186/s12864-022-09077-4

**Published:** 2022-12-15

**Authors:** Hui Jiang, Zhi-Xin Chai, Han-Wen Cao, Cheng-Fu Zhang, Yong Zhu, Qiang Zhang, Jin-Wei Xin

**Affiliations:** 1 State Key Laboratory of Hulless Barley and Yak Germplasm Resources and Genetic Improvement, Lhasa, 850000 Tibet China; 2 Key Laboratory of Animal Genetics and Breeding on Tibetan Plateau, Ministry of Agriculture and Rural Affairs, Institute of Animal Science and Veterinary (TAAAS), Lhasa, 850009 Tibet China; 3 Key Laboratory of Qinghai-Tibetan Plateau Animal Genetic Resource Reservation and Utilization, Sichuan Province and Ministry of Education, Southwest Minzu University, Chengdu, 610041 Sichuan China

**Keywords:** Adrenomedullin, Body weight, Parathyroid hormone, SNP chip, Yak

## Abstract

**Background:**

The yak is the most important livestock in the Qinghai-Tibet Plateau, and body weight directly affects the economic values of yak. Up to date, the genome-wide profiling of single-nucleotide polymorphisms (SNPs) associating with body weight has not been reported in yak. In the present study, the SNPs in 480 yaks from three breeds were analyzed using the commercial high-density (600 K) yak SNP chips.

**Results:**

The results identified 12 and 4 SNPs potentially associated with body weight in male and female yaks, respectively. Among them, 9 and 2 SNPs showed significant difference in yak body weight between different genotypes at each locus in male and female yaks, respectively. Further exploration found 33 coding genes within the 100 kbp upstream or downstream to the SNP loci, which might be potentially affected by the variation of SNPs. Among them, G protein-coupled receptor kinase 4 (GRK4) might be potentially affected by the SNP AX-174555047, which has been reported to affect the functioning of two body-weight associated hormones (parathyroid hormone, PTH, and adrenomedullin, ADM). Determination of PTH and ADM levels in yak revealed positive relationship between PTH level and body weight, negative relationship between ADM level and body weight along with the variation of AX-174555047 mutation.

**Conclusions:**

These results suggested that the SNP AX-174555047 might potentially affect body weight through mediating GRK4 expression and then PTH and ADM functioning. Thus, the SNP AX-174555047 might be used as a biomarker for molecular breeding of yak. More investigations are required to validate this point.

**Supplementary Information:**

The online version contains supplementary material available at 10.1186/s12864-022-09077-4.

## Background

The yak (Bovidae: *Bos grunniens*) is naturally distributed across the Qinghai-Tibetan Plateau (QTP) and adjacent highlands, and provides meat, hides, dung fuel and transport power to the local residents. Breeding of yak aims to obtain strains with high-quality growth traits, such as high body weight, growth speed and meat quality [1]. The traditional breeding technique requires cross-hybridization between individuals from various strains, which is time-consuming and highly costs. Alternatively, molecular breeding could rapidly identify parent animals with high-quality traits, which requires screening of trait-associated markers as the fundamental basis.

At early time, microsatellite (SSR) was the commonly used molecular breeding markers. Using the SSR technology, Cai et al. [2] and Asma et al. [3] identified 92 and 30 SSR loci in sampled breeding populations of Maiwa yak, respectively, and the authors claimed that these results might contribute importantly to the molecular breeding of yak. Compared with the SSR technology, single nucleotide polymorphism (SNP) technology could provide more detailed and precise information to understand the gene mutations and the affected biological functions. In yak, Cai et al. [4] found five SNPs in the coding region of the melanocortin receptor 4 (MC4R) gene, and one of them (SNP4: 1069G > C) may associate with the increased feed intake, body weight and average daily gain of the yaks. Wu et al. [5] identified three SNPs in the region of endothelial PAS domain protein 1 gene (EPAS1), among which, the SNP g.83065 G > A was highly associated with hemoglobin concentration in yak. These results directly offered molecular markers for breeding of yak of good growth performance. However, these two studies only focused on certain gene regions. Moreover, a few studies have reported genome-wide mutations in yak. For example, from 16 yak populations, Wang et al*.* [6] detected 51,461 copy number variations (CNVs) and 3174 copy number variation regions (CNVRs), and further analyzed their potential effects on immune response, glucose metabolism, sensory perception, and adaptation to hypoxia. By comparison of genome sequences between 50 polled yaks and 51 horned yaks, a 147-kb segment that included three protein-coding genes *C1H21orf62*, *GCFC1* and *SYNJ1* was the most likely location of the poll mutation in domestic yaks [7]. However, to the best of our knowledge, genome-wide detection of SNPs in yak has not been reported yet.

Compared with high-throughput genome sequencing, gene chip is low cost, more accurate, and convenient to operate, and has been widely used in breeding. Based on the gene chip technique, dense SNP genotyping arrays (DSNPGA), which incorporate thousands of SNP loci in a single chip for testing, provide extensive information on polymorphic variations across the genome of species of interest. Such information can be used in studies of selective sweeps, phylogeny, population structure, copy number variations, and genome-wide association analysis (GWAS). Based on DSNPGA and GWAS, multiple genes related to little size trait in Pelibuey sheep [8], four candidate genes associated with body weight in Baluchi sheep [9], two genes associated with carcass weight in pig [10], and 105 SNPs potentially affecting milk production traits in Chinese Holstein population have been detected [11]. In yaks, Fu et al. [12] genotyped yak SNP using an Illumina Bovine HD 770 K chip. However, the obtained results were not informative enough, since the used chip was designed against bovine genome, and there are great differences between yak and bovine genomes. Therefore, incorporating abundant specific SNPs of yak are still required for subsequent high-quality GWAS.

Body weight is an important index to evaluate meat production performance [13, 14]. In this study, based on the commercial yak high-density 600 K SNP chip, the variations of SNPs were examined in 480 yak individuals, and GWAS was conducted to screen SNPs potentially affecting yak body weight. Afterwards, the biological functions potentially affected by SNPs were predicted, and validated by determining serum levels of metabolites. These results would provide novel information of SNPs for future molecular breeding of yak.

## Results and Discussion

### Identification of SNPs and genetic differences between three yak populations

A total of 480 individuals were analyzed using the yak high-density 600 K SNP Chip. Finally, 13 individuals were discarded since their Dish QC values (an indicator representing the difference between signal value of the reaction probe and the background) were less than 0.82, and 467 individuals passed the quality control. Further genotyping analysis revealed that the average call rate of the 467 individuals was 0.9615. The average number of SNP passing the quality control was 605,946.

**Fig. 1:**
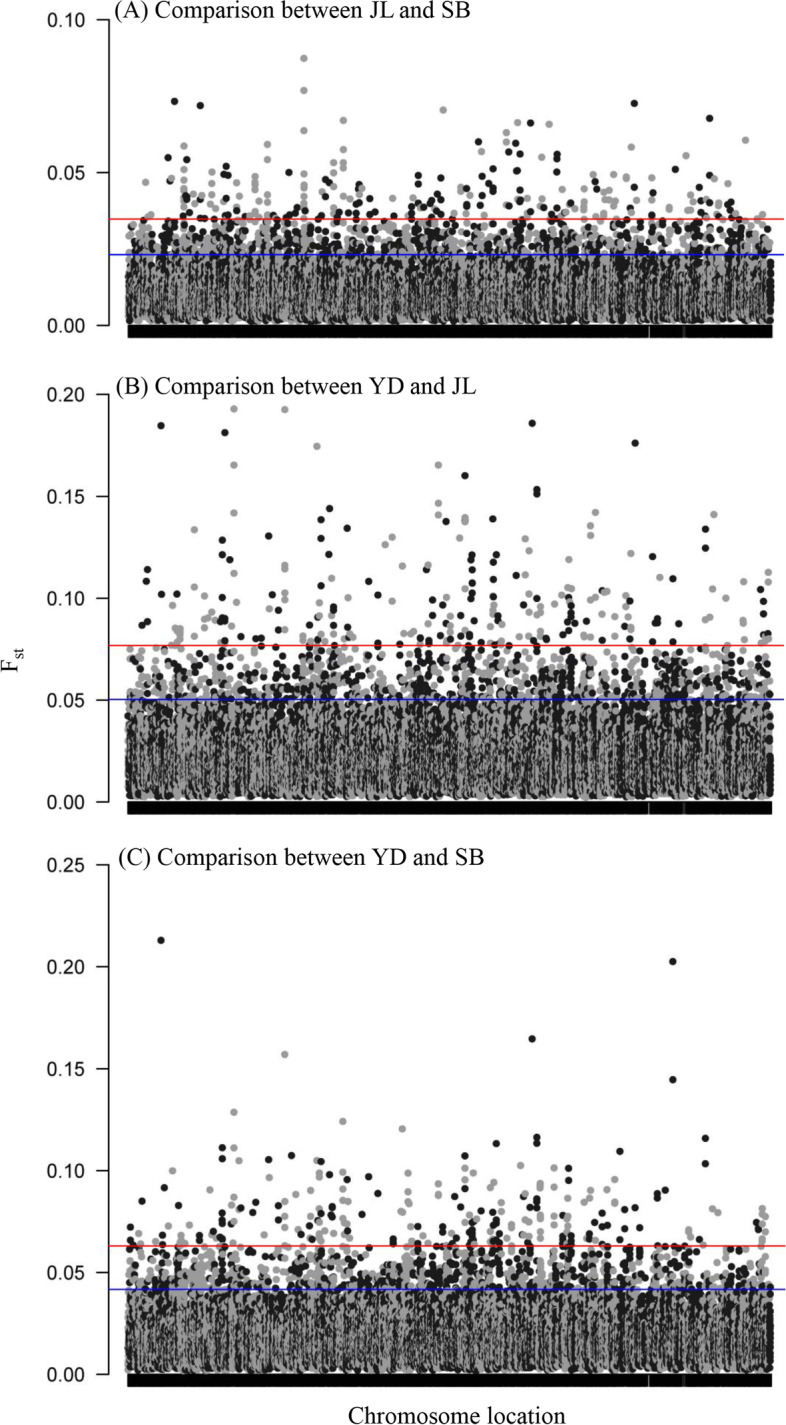
Genetic differentiation of SNP loci between three yak populations (JL, SB and YD)

**Fig. 2:**
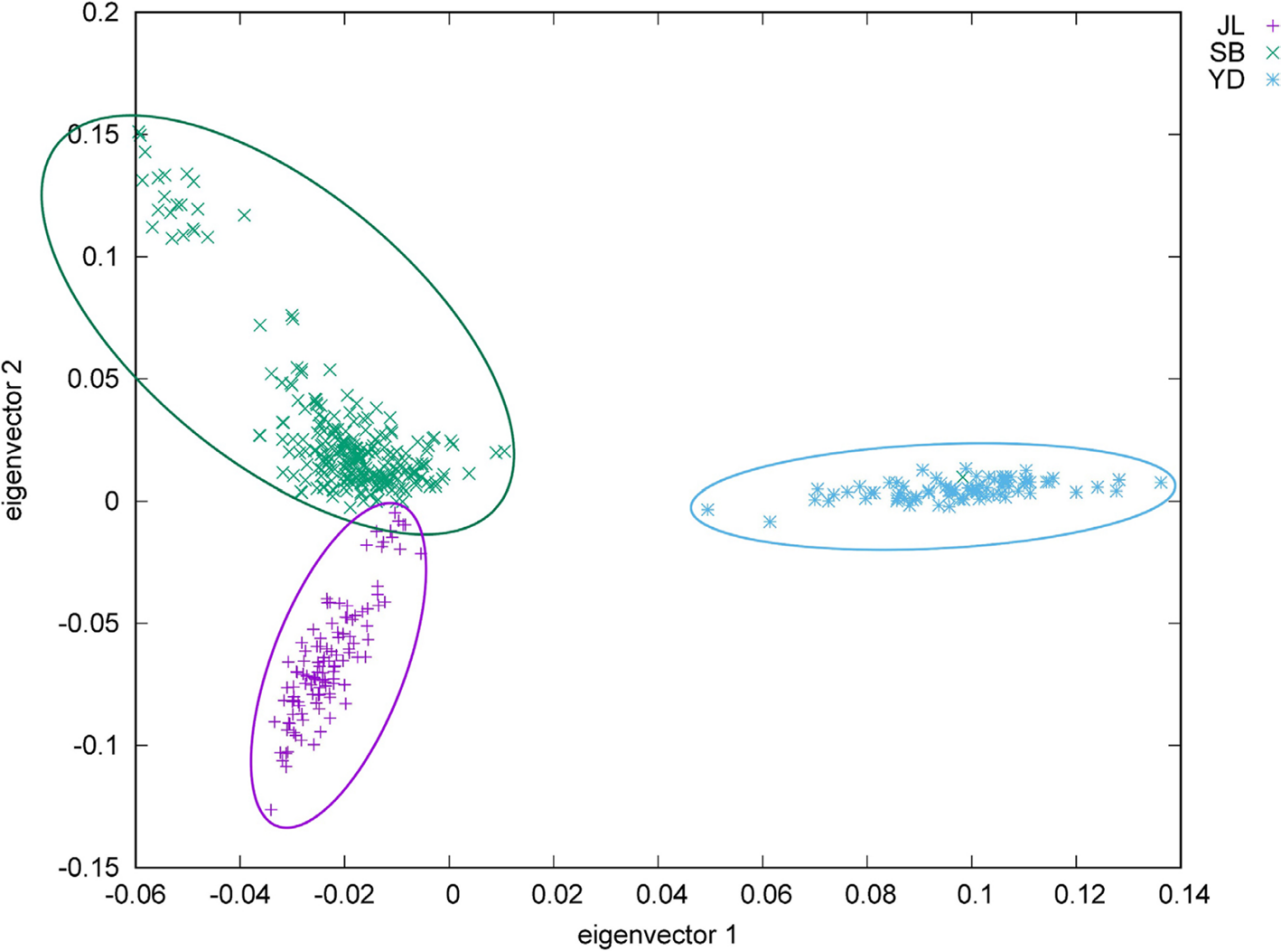
PCA analysis between three yak populations (JL, SB and YD) based the variations of SNP loci

Genetic differences (F_st_) at each SNP locus between Jiali yak (JL), Pali yak (YD) and Sibu yak (SB) yak populations are shown in Fig. 1 (details in Supplementary Table S 1). The F_st_ values between YD and JL, YD and SB were relatively higher than that between JL and SB (Fig. 1C). Consistent with the F_st_ results, PCA analysis indicated that YD, JL and YD separated well on the plot (Fig. 2), suggesting a high level of genetic differentiation between yak populations.

**Fig. 3:**
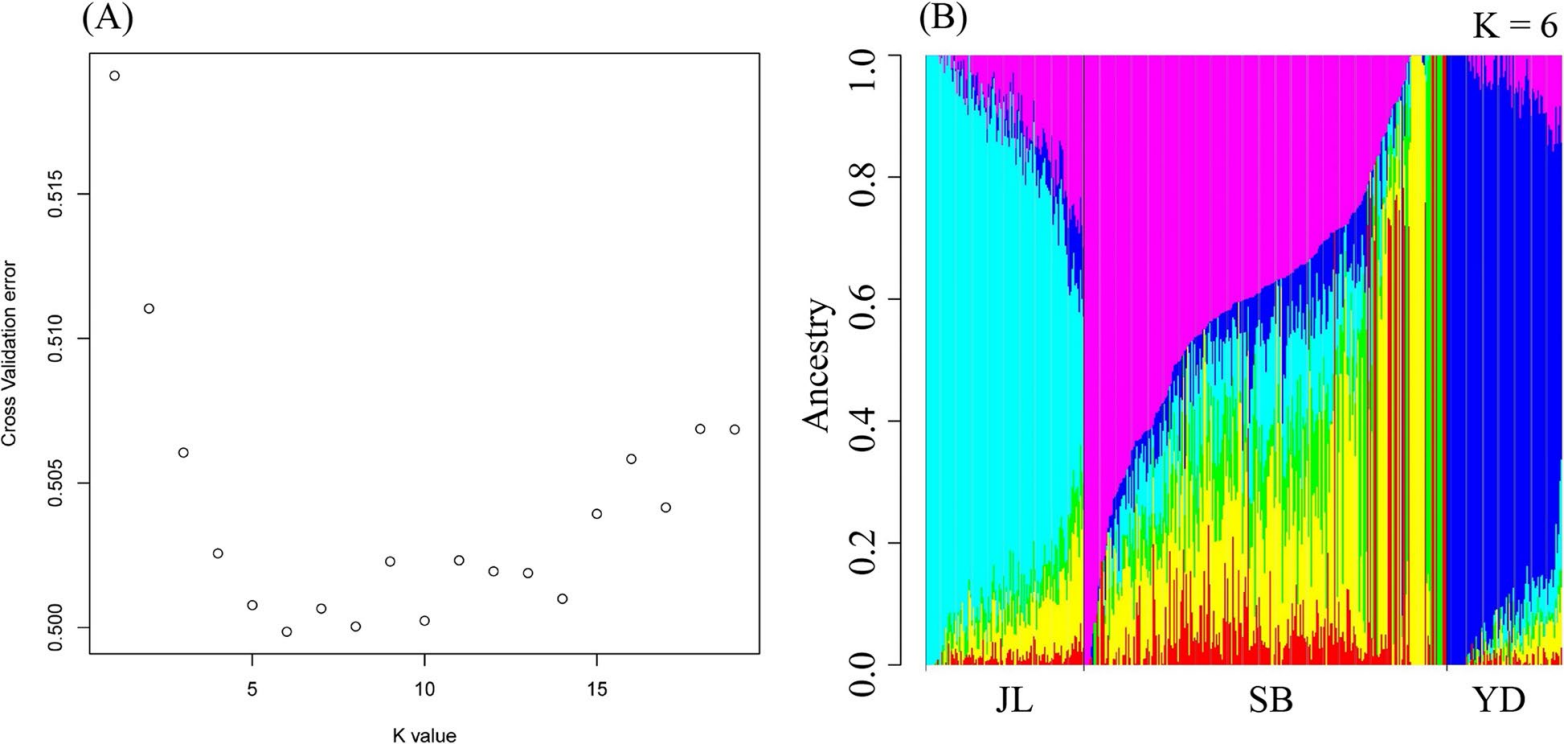
Admixture analysis of SNPs from three yak populations (JL, SB and YD). **A**. Cross-validation errors at different ancestry number. **B**. Proportions of individual-level ancestries in JL, SB and YD yaks when the putative ancestry number was six

Results of Admixture analyses are showed in Fig. 3. The cross validation showed the lowest error at the ancestry number of 6 (Fig. 3A), suggesting that yaks analyzed in this study might have 6 putative ancestries. Under this setting, JL and YD populations showed relatively higher proportions of one putative ancestry than SB population, but SB population had a higher level of ancestry diversity than other two populations (Fig. 3B).

### Screening of SNPs associated with yak body weight

**Fig. 4:**
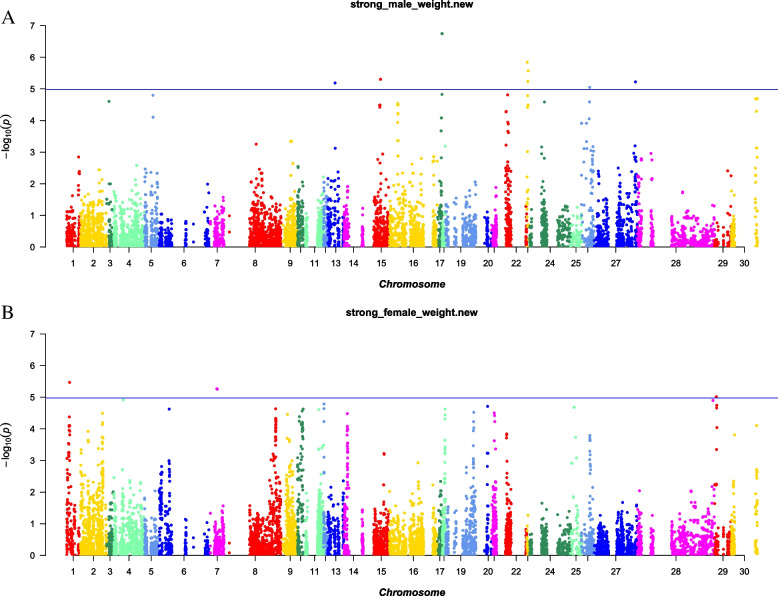
Manhattan plot showing single-nucleotide polymorphisms (SNPs) associated with body weight of male and female on the yak chromosome. **A**. male **B**. female

**Fig. 5:**
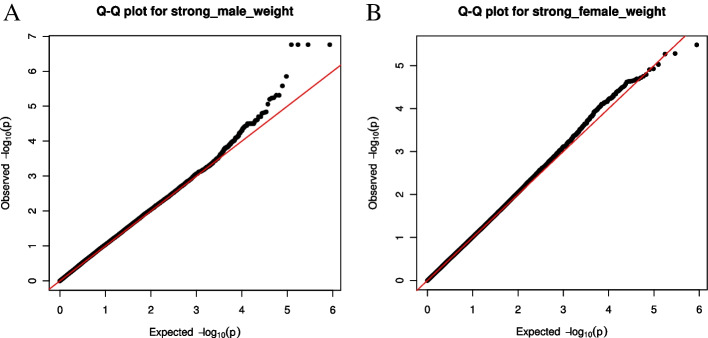
Quantile–quantile plot of genome-wide association study (GWAS) shown in the Manhattan plot. **A**. male **B**. female

Body weight-associated SNPs have not been reported in yaks, but have been reported in other livestock. For instance, in the Landrace × Korean native pig, 5 SNPs at the LOC100621652 and LOC100523510 genes on the chromosome 12 were identified to highly associate with carcass weight using the 60 K Porcine SNP chips [10]. In the Baluchi sheep, 13 SNPs were identified to associate with body weight using the Ovine 50 K SNP chip [15]. In the present study, considering the great inherent difference in body weight between male and female yaks, the GWAS were conducted separately in male and female animals. After correction using the BH method, only four SNPs were significantly associated with body weight in male yak (FDR < 0.05), and none SNPs were significantly associated with body weight in female yak (Fig. 4 and Table 1). To expand the scope of screening, the screening threshold was adjusted to the uncorrected P value of 1.0 × 10^–6^, based on which, 12 and 4 SNP loci significantly associated with body weight were identified in male and female yaks, respectively (Fig. 4 and Table 1). The quantile–quantile (Q-Q) plots showed these significant SNPs were deviated between observed and expected p-values with the genomic inflation factor (λ) values of 1.09 and 1.01, respectively (Fig. 5), suggesting selection on these SNPs. The percentage of the phenotypic variance explained (PVE) (R^2^) by the significant SNPs was shown in Table 1. The PVE of AX-174734142 and AX-174706158 was 7.4%, suggesting that these two SNPs revealed great influences on yak body weight.

**Table 1:** GWAS analyses identified 16 SNP loci significantly associated with body weight in male and female yaks

CHR	Physical Position	SNP	P	FDR_BH	Allele A	Allele B	BETA	R^2^(%)
Male								
scaffold2344_1	326,970	AX-174702570	1.80E-07	0.02	A	G	67.9	5.59
scaffold2344_1	327,739	AX-174961896	1.80E-07	0.02	T	G	67.9	5.59
scaffold2344_1	337,514	AX-174407967	1.80E-07	0.02	A	G	67.9	5.59
scaffold2344_1	337,544	AX-174402854	1.80E-07	0.02	A	G	67.9	5.59
scaffold4472_1	23,932	AX-174929694	1.43E-06	0.12	A	G	41.84	4.00
scaffold4472_1	82,966	AX-174547362	2.68E-06	0.20	A	G	45.29	5.25
scaffold2104_1	1,168,770	AX-174734142	4.97E-06	0.25	A	G	-48.47	7.40
scaffold2104_1	1,170,706	AX-174706158	4.97E-06	0.25	A	G	-48.47	7.40
scaffold4472_1	68,096	AX-174783962	5.82E-06	0.25	A	G	43.11	5.41
scaffold70_3	2,788,947	AX-174627015	5.98E-06	0.25	A	G	51.08	8.14
scaffold2021_1	491,659	AX-174928167	6.47E-06	0.25	T	C	49.71	0.27
scaffold699_1	549,280	AX-174555047	8.94E-06	0.32	T	G	-50.34	0.26
Female								
scaffold10_1	223,744	AX-174845027	3.39E-06	0.56	A	G	-19.78	2.10
scaffold1366_1	539,922	AX-174891371	5.39E-06	0.56	T	C	68.75	0.87
scaffold1366_1	561,203	AX-174570649	5.56E-06	0.56	A	G	68.94	0.29
scaffold923_1	123,435	AX-174620133	9.60E-06	0.56	T	C	-19.78	2.72

*FDR* False discovery rate

R^2^: phenotypic variance explained (PVE) by the significant SNP

The results of linkage disequilibrium (LD) analysis between significant SNPs were shown in Table 2. The D’ of AX-174702570, AX-174961896, AX-174407967 and AX-174402854 were 1.0, suggesting complete co-transferring in Scaffold2344_1. AX-174929694, AX-174547362, and AX-174783962 completely co-transferred in Scaffold4472_1; AX-174734142 and AX-174706158 completely co-transferred in scaffold2104_1; AX-174891371and AX-174570649 completely co-transferred in scaffold1366_1. However, AX-174627015 in scaffold70_3, AX-174928167 in scaffold2021_1, AX-174555047 in scaffold699_1, AX-174845027 in scaffold10_1, and AX-174620133 in scaffold923_1 were independent, and did not link with other significant SNPs in the corresponding regions. These results indicated that only five of the 16 SNPs significantly associated with yak body weight were independently inherited.

**Table 2:** Linkage disequilibrium of 16 SNPs genotype

L1	L2	CHR	D’	r^2^
AX-174702570	AX-174961896	scaffold2344_1	1.0	1.0
	AX-174407967	scaffold2344_1	1.0	1.0
	AX-174402854	scaffold2344_1	1.0	1.0
AX-174961896	AX-174407967	scaffold2344_1	1.0	1.0
	AX-174402854	scaffold2344_1	1.0	1.0
AX-174407967	AX-174402854	scaffold2344_1	1.0	1.0
AX-174929694	AX-174783962	scaffold4472_1	0.87	0.62
	AX-174547362	scaffold4472_1	0.88	0.63
AX-174783962	AX-174547362	scaffold4472_1	0.99	0.97
AX-174734142	AX-174706158	scaffold2104_1	1.0	1.0
AX-174627015	/	scaffold70_3	/	/
AX-174928167	/	scaffold2021_1	/	/
AX-174555047	/	scaffold699_1	/	/
AX-174845027	/	scaffold10_1	/	/
AX-174891371	AX-174570649	scaffold1366_1	1.0	0.85
AX-174620133	/	scaffold923_1	/	/

**/:** There is no linkage disequilibrium site in this region

### Comparison of yak body weight between different genotypes at each locus

**Fig. 6:**
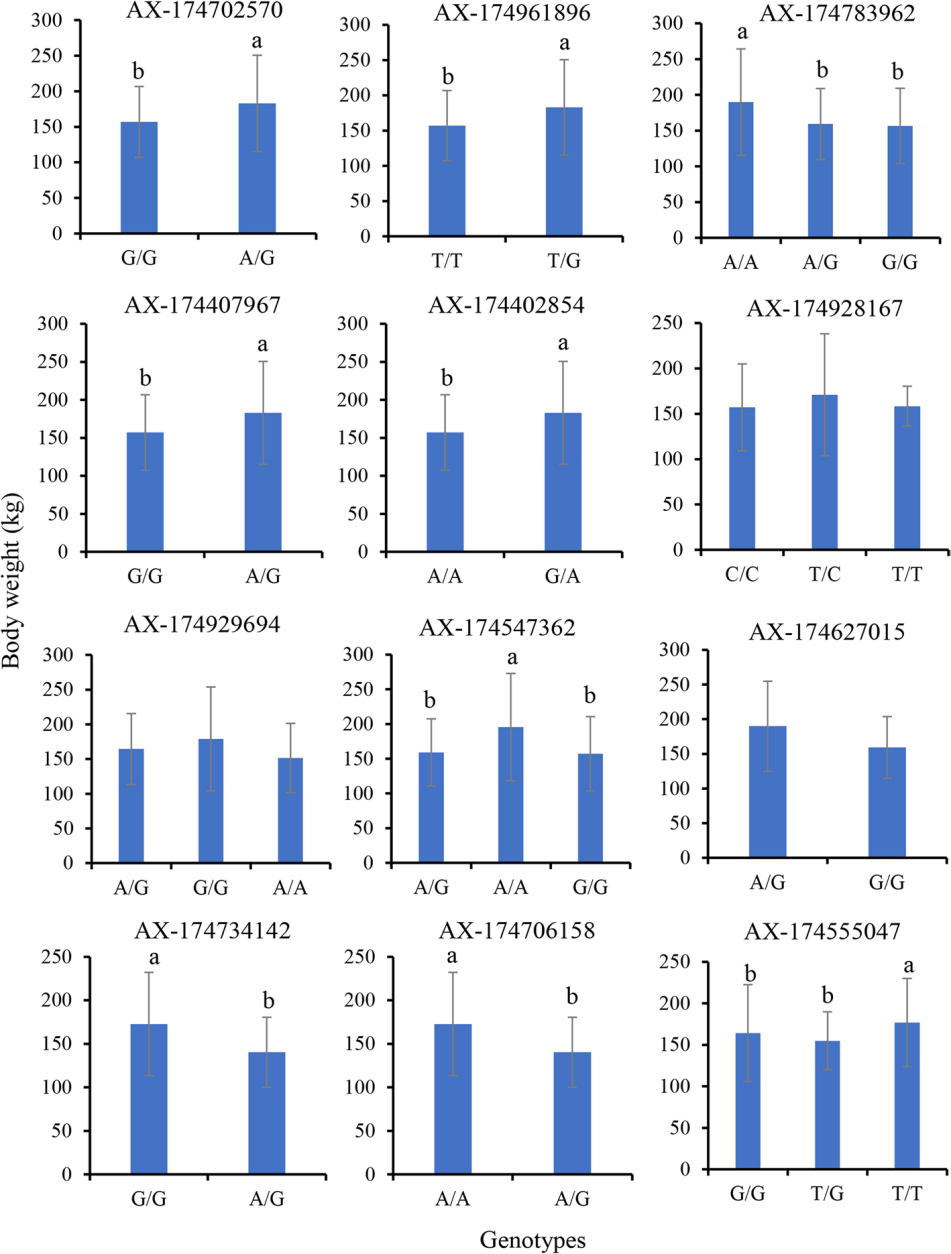
Comparison of body weight between different genotypes at 12 SNP loci (mean ± SD) in male yaks. Different letters above the bars indicate significant difference between genotypes at each locus (*P* < 0.05)

In male yaks, 9 of the 12 selected SNPs showed significantly different body weight between different genotypes. Among them, A/G type at AX-174702570, T/G type at AX-174961896, A/G type at AX-174407967, G/A type at AX-174402854, G/G type at AX-174734142, and A/A type at AX-174706158 revealed significantly higher body weight than the G/G, T/T, G/G, A/A, A/G and A/G type, respectively. In addition, the A/A type at AX-174783962 displayed significantly higher body weight than A/G and G/G types. A/A type indicated significantly body weight than A/G and G/G types at AX-174547362. At AX-174555047, T/T type showed significantly higher body weight than G/G and T/G types (Fig. 6).

**Fig. 7:**
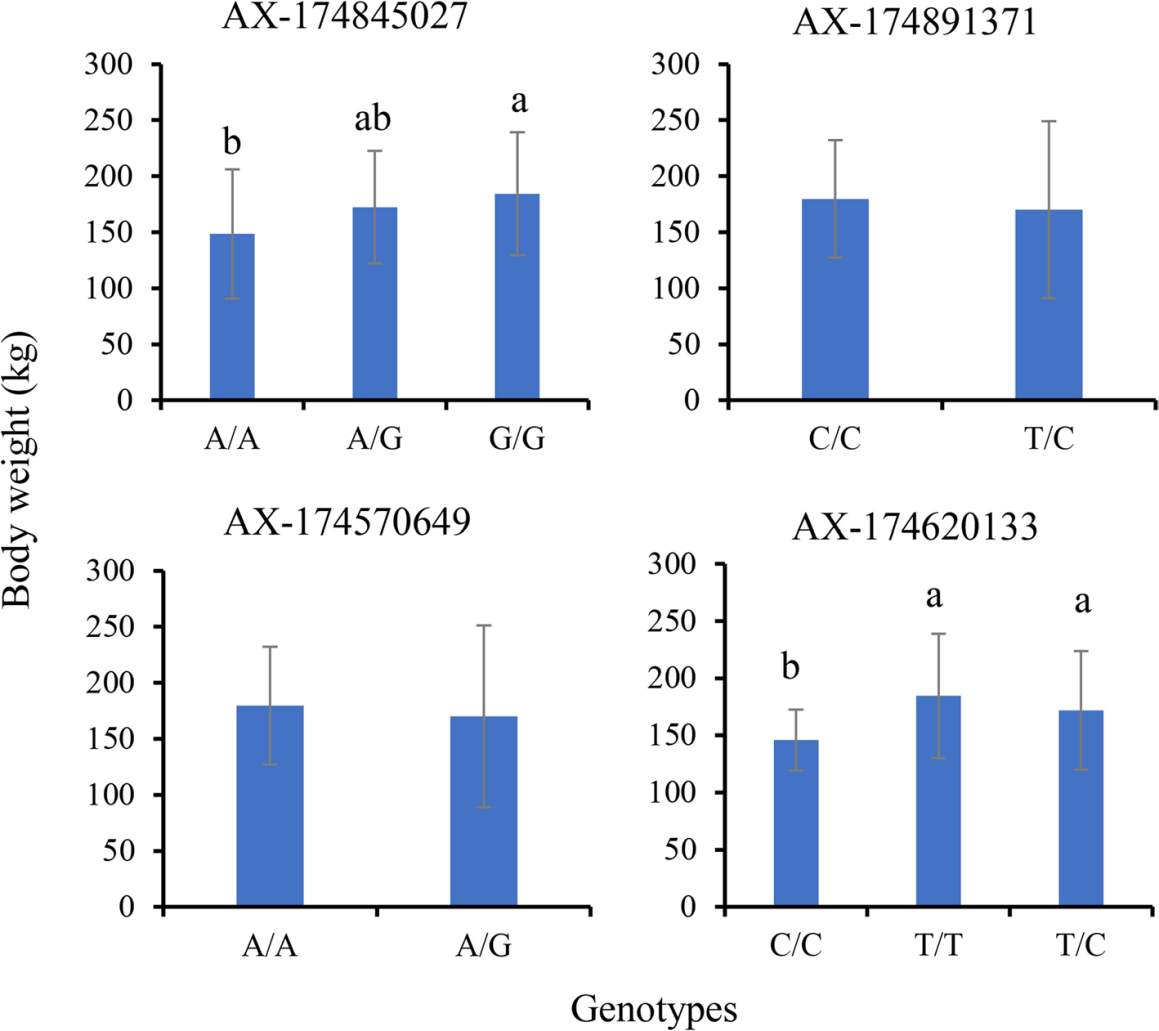
Comparison of body weight between different genotypes at four SNP loci (mean ± SD) in female yaks. Different letters above the bars indicate significant difference between genotypes at each locus (*P* < 0.05)

In female yaks, two of the four selected SNPs showed significantly different body weight between genotypes. Among them, G/G showed significantly higher body weight than A/G type at AX-174845027, but both of them did not significantly differ from the A/G type. At AX-174620133, the T/T and T/C types revealed significantly higher body weight than the C/C type (Fig. 7).

### Identification of genes potentially affected by the variation of the 11 SNPs

A total of 11 SNPs were identified to affect body weight in yak, which were localized to six scaffolds (Table 3). Within the distance of 100 kb from the SNPs, 33 coding genes were obtained, which were speculated to be potentially affected by the 11 SNPs. The SNPs AX-174702570, AX-174961896, AX-174407967 and AX-174402854 were rather close in the scaffold2344_1, and they might affect the same gene cluster, including family with sequence similarity 183 member A (FAM183A), EBNA1 binding protein 2 (EBNA1BP2), and cilia and flagella associated protein 57 (CFAP57). Similarly, the SNPs AX-174783962 and AX174547362 were close in the scaffold4472_1, and they might affect Rho GTPase-activating protein 19 (ARHGAP19). The SNPs AX-174555047, AX-174734142, AX-174620133 and AX-174845027 were localized to the scaffold699_1, scaffold2104_1, scaffold923_1 and scaffold10_1, and potentially influenced 11, 2, 7 and 9 genes, respectively (Table 3).

**Table 3:** 33 genes potentially affected by the 11 SNP loci

Chrome	Position	SNP	Potentially affected genes
Male yaks			
scaffold2344_1	326,970 327,739 337,514 337,544	AX-174702570 AX-174961896 AX-174407967 AX-174402854	FAM183A, EBNA1BP2, CFAP57
scaffold699_1	549,280	AX-174555047	CFAP99, RNF4, FAM193A, TNIP2, SH3BP2, ADD1, MFSD10, NOP14, GRK4, HTT, MSANTD1
scaffold4472_1	68,096 82,966	AX-174783962 AX-174547362	ARHGAP19
scaffold2104_1	1,168,770	AX-174734142	OPCML, NTM
Female yaks			
scaffold923_1	123,435	AX-174620133	ERP44, STX17, NR4A3, SEC61B, ALG2, TGFBR1, COL15A1
scaffold10_1	223,744	AX-174845027	SSX2IP, CTBS, SPATA1, GNG5, RPF1, DNASE2B, SAMD13, PRKACB, TTLL7

Among these 33 genes, ARHGAP19, SH3 domain-binding protein 2 (SH3BP2), nucleolar protein 14 (NOP14), afadin- and alpha-actinin-binding protein (SSX2IP) and cAMP-dependent protein kinase catalytic subunit beta (PRKACB) were related to cell proliferation, migration and differentiation. G protein-coupled receptor kinase 4 (GRK4) has been reported to associate with obesity and muscular dystrophy [16, 17]. Nuclear receptor subfamily 4 group A member 3 (NR4A3) and COL15A1 (COL15A1) are muscle regulatory factors. NR4A3 can regulate the expression of small muscular protein (SMPX) in muscle cells and promote myotube differentiation, which directly affects muscle formation [18]. COL15A1 is a basement membrane component mainly expressing in skeletal muscle, and plays a direct regulatory role in muscle development [19]. These genes deemed further investigations in yak to demonstrate whether the selected SNPs could affect their expression and then further influence body weight. Unfortunately, due to the lack of yak tissue samples, we could not explore the mRNA or protein levels in differentially genotyped yaks. In future, we will try to collect yak tissue samples to validate these hypotheses.

### Comparisons of parathyroid hormone (PTH) and adrenomedullin (ADM) levels in yaks with different genotypes at the AX-174555047 locus

**Fig. 8:**
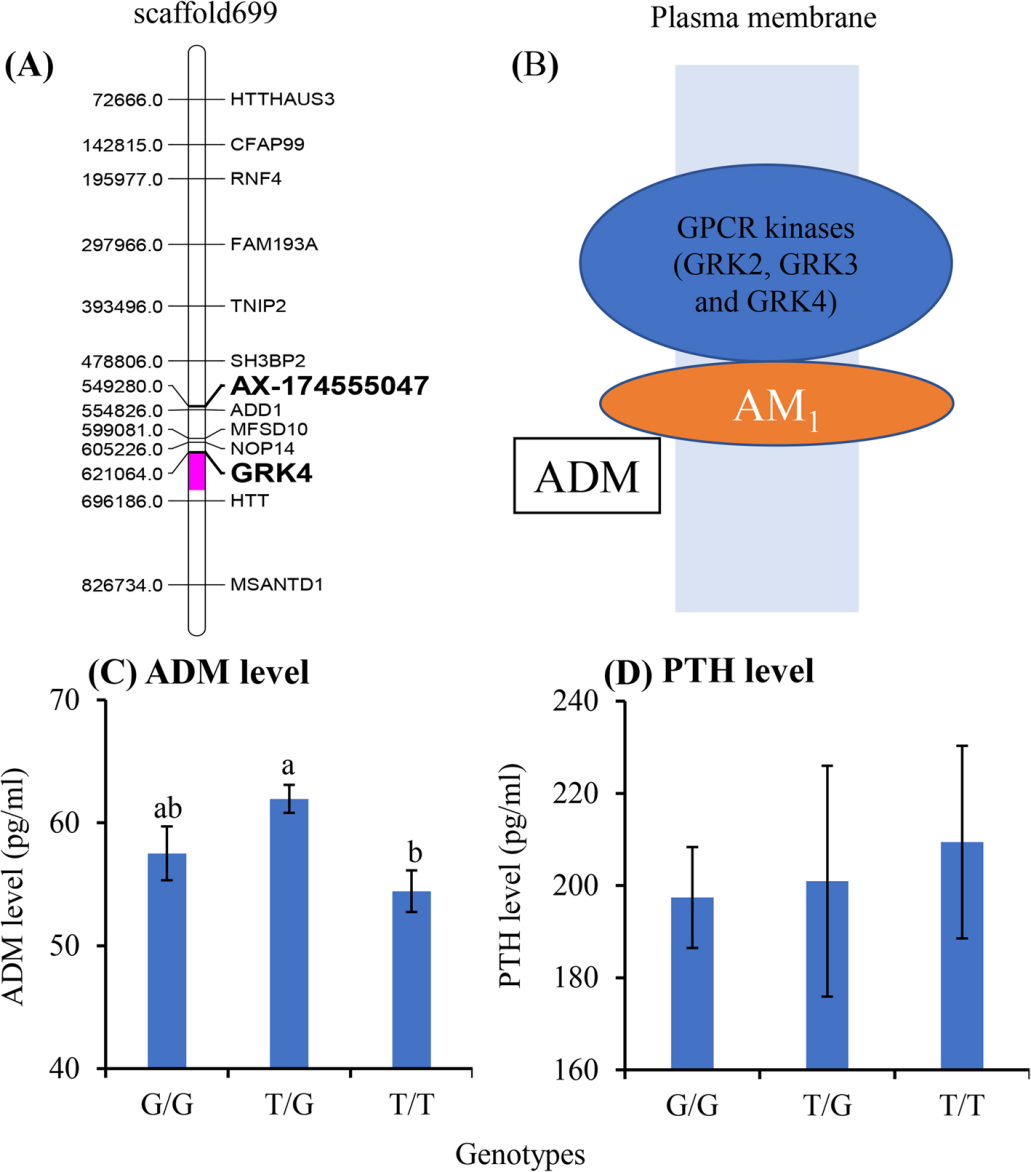
The SNP AX-17455504 might affect ADM and PTH levels through mediating GRK4 gene. **A**. The location of AX-17455504 and GRK4 gene on the scaffold699. **B**. Illustration of the relationship between AMD GRK4. GRK4: G protein coupled receptor kinase 4; ADM: adrenomedullin; AM_1_: ADM receptor. **C** AMD levels between different genotypes at the SNP AX-17455504. **D** PTH levels between different genotypes at the SNP AX-17455504

The SNP locus AX-174555047 was localized to the scaffold699_1, and might potentially affect the downstream GRK4 gene (Fig. 8A). GRK4 was reported to regulate the functioning of ADM and PTH, which are two important hormones affecting body weight [20, 21]. As reported, ADM is a kind of bioactive peptide produced by adipose tissue and promoting angiogenesis and proliferation of preadipocytes. Generally, obese people show higher ADM levels than non-obese people [22, 23]. In addition, GRK4 negatively regulates ADM functioning by inhibiting cell surface expression and signal transduction of ADM receptor (AM_1_) [21]. The overexpression of GRK4 resulted in a marked impairment of the Gs coupling and/or activation in the ADM receptor (Fig. 8B) [21, 24]. In the present study, for the AX-174555047 SNP locus, the T/G genotype showed higher ADM level than G/G, and then than T/T genotype, with significant difference between T/G and T/T types (Fig. 8C). Differently, the T/G genotype showed lower body weight than G/G, and then than T/T genotype (Fig. 6). These inverse trends (*r* = -0.21, Table S 3) between body weight and ADM level along with change of genotype suggested that variation of AX-174555047 SNP locus might regulate the body weight through meditating the functioning of ADM-ADM receptor system.

It has bene reported that body mass index (BMI) was significantly positively correlated with PTH level [22], and obesity was associated with increased PTH levels in chronic disease patients [25]. In addition, GRKs regulated the responses of PTH receptor [20]. In this study, the T/T type showed significantly higher body weight but insignificantly higher PTH level than the G/G and T/G types (Figs. 6 and 8D). These results neither effectively supported nor denied the roles of AX-174555047 SNP locus in variation of yak body weight. Since the PTH levels in each genotype showed a great standard deviation, more samples were required to further validate the relationship of AX-174555047 mutation, PTH level, PTH functioning and body weight in yak.

## Conclusions

GWAS identified 12 and 4 SNPs potentially associated with body weight in male and female yaks, respectively. The SNP AX-174555047 might regulate body weight through affecting PTH and ADM functioning by mediating the GRK4 gene in yak, and might be used as a biomarker for molecular breeding of yak in future.

## Materials and methods

### Sample collection

In the present study, a total of 480 yaks aged from 4 to 9 years with the body weight lower than 90 kg or higher than 130 kg were included. These yaks were from three yak populations (JL yak, YD yak and SB yak). The information of sampling locations is shown in Table 4. Venous blood was collected from each yak for DNA extraction.

**Table 4:** The location information of yaks used in the present study

Breed	Location	Longitude and latitude	Altitude (m)
Jiali yak	Tibet, Lhari County	30°69’ N, 93°32’ E	4488
Pali yak	Tibet, Yadong County	27°72’ N, 89°16’ E	2945
Sibu yak	Tibet, Maizhokunggar County	29°71’ N, 91°87’ E	3835

### DNA extraction and SNP genotyping

For each yak, DNA was extracted from 200 μl of blood using the TIANamp Genomic DNA kit (Tiangen, Beijing, China) according to the manufacturer’s protocol. The concentration of DNA was quantified using a NanoDrop ND-2000 spectrophotometer (Thermo Fisher Scientific Inc., Waltham, MA USA) and the DNA integrity was assessed by 1% agarose gel electrophoresis.

SNP loci were determined using the commercial Yak high-density 600 K SNP Chip (630,209 SNP) produced by the Affymetrix company (Santa Clara, CA, USA) and the Axiom 2.0 reagent kit (ThermoFisher, USA). The speciation of the Yak high-density 600 K SNP Chip is presented in Supplementary file 1 and information of each locus is shown in Supplementary Table S 2. Briefly, the DNA concentration of each sample was adjusted to 10 ng/μl, and then 20 μl of DNA solution was mixed with 20 μl of Denaturation Master Mix for denaturation. Next, 130 μl of Axiom 2.0 Neutral Solution and 230 μl of Amplification Master Mix were added. The mixture was amplified at 37℃ for 23 h, and then the reaction was stopped by heating at 65℃ for 30 min. Afterwards, the amplified products were fragmented by adding 57 μl of Fragmentation Master Mix and then incubation at 37℃ for 30 min. The fragmentation reaction was terminated by adding 19 μl of Axiom Frag Rxn stop solution. After mixed with 240 μl of Precipitation Master Mix solution and 600 μl of isopropanol at -20 ℃ for 24 h, the solutions were centrifugated at 3,200 g for 40 min. The precipitated DNA pellets were dried at 37℃ for 20 min in a GeneChip Hybridization Oven 645, redissolved in 35 μl of Axiom Resusp Buffer, and mixed with 80 μl of Hybridization Master Mix solution. The quantity and quality of DNA fragments were inspected using a NanoDrop ND-2000 spectrophotometer and an Agilent 2100 bioanalyzer (Agilent Technologies, Palo Alto, Calif.). Samples with DNA concentration higher than 100 ng/μl and fragment size between 25 and 125 bp were considered qualified.

The hybridization plates were placed in a PCR instrument for denaturation. The denaturation program included incubation at 95℃ for 10 min, 48℃ for 3 min and finally 48℃ for hold. Next, the denatured samples were transferred from the Hybridization Ready plate into the Hybridization tray, and the hybridization was conducted using a GeneTitan MC Instrument for 24 h. The Stain, Ligation and Stabilization mix solutions were added for staining. Finally, the hybridization chips were scanned using a Genetitian Multi-Channel (MC) Instrument (Thermo Fisher), and analyzed using the GeneTitan software to obtain the SNP calls of each sample.

The SNP calls were exported to Axiom Analysis Suite for genotype calling. Next, quality control was carried out using the PLINK program [26]. SNPs with the following criteria were removed: (1) minor allele frequencies (MAF) < 0.01, (2) call rate < 0.95, and/or (3) Hardy–Weinberg equilibrium (HWE) < 0.001.

The F_st_ values of each SNP were calculated by the Bayesian method [27]. A 100-kb sliding window with 50-kb step was applied to calculate the average F_st_ value along all chromosomes. The EIGENSOFT (v6.0.1) software [28] was used for PCA analysis to obtain the first four principal components with the most genetic variance. ADMIXTURE (v1.23) software [29] was used to analyze the population structure, detecting the number of ancestors with the smallest cross validation error.

The linkage disequilibrium (LD) between candidate SNPs were analyzed using the LDBlockShow software (v1.40) [30]. To quantify LD, widely used D’ and r^2^ were calculated. A value of zero implies independence, whereas 1.0 means complete co-transferring. Phenotypic variance explained (PVE) of each SNP was calculated using the PLINK software [26].

### Selection of coding genes potentially affected by SNPs

Based on the yak genome sequence [31], the chromosomal locations of the SNPs were retrieved, and coding genes within 100 kilobases upstream or downstream to SNPs [32] were selected as the target genes potentially affected by SNPs.

### Measurement of serum contents of bovine parathyroid hormone (PTH) and adrenomedullin (ADM)

TO validate the biological functions potentially affected by variation of the AX-174555047 SNP locus, PTH and ADM contents in yak sera were detected in 93 yaks, which were collected from another batch of yak samples, including Chawula yak, Jila yak, Leiwuqi yak and Shenzha yak, using enzyme linked immunosorbent assay (ELISA) kits produced by the Jingmei Technology Company (Jiangsu, China) following the manufacturer’s protocols. The sample sizes of the T/T, T/G and G/G genotypes at the AX-174555047 locus were 3, 53 and 37 individuals, respectively.

### Statistical analysis

GWAS was performed using the mix linear model (MLM) and the GAPIT software [26]. The first three components of PCA analysis and gender were included as the covariables. For each locus, the *p* value was corrected using the Benjamini-Hochberg (BH) method by setting the false discovery rate (FDR) < 0.05. However, following this criterion, only four significant SNPs were identified. To expand the screening scope, we manually reset the screening threshold using the uncorrected P value of 1.0 × 10^–5^. At the uncorrected P value of 1.0 × 10^–5^, the Q-Q plot revealed that the observed value started to greatly deviate from the predicted value.

Differences in body weight, PTH and AMD levels were compared between different genotypes at each locus using one-way ANOVA (for SNPs having three genotypes) or Student’s *t*-test (for SNPs having two genotypes). Values were considered significantly different with the *P* < 0.05.

## Supplementary Information

**Additional file 1.**

**Additional file 2.**

**Additional file 3.**

**Additional file 4.**

## Data Availability

The yak genome sequences are deposited to NCBI under accession number PRJNA670822. The genome-wide SNP and phenotypic datasets of yak are available in the Figshare repository (https://doi.org/10.6084/m9.figshare.9408278). The speciation of the Yak high-density 600 K SNP Chip is presented in Supplementary file 1 and information of each locus is shown in Supplementary Table S 2.

## Abbreviations

ADM: Adrenomedullin
ARHGAP19: Rho GTPase-activating protein 19
CFAP57: Cilia and flagella associated protein 57
CNVs: Copy number variations
CNVRs: Copy number variation regions
DSNPGA: Dense SNP genotyping arrays
EBNA1BP2: EBNA1 binding protein 2
EPAS1: Endothelial PAS domain protein 1 gene
FAM183A: Family with sequence similarity 183 member A
GWAS: Genome-wide association analysis
MC4R: Melanocortin receptor 4
NR4A3: Nuclear receptor subfamily 4 group A member 3
PRKACB: CAMP-dependent protein kinase catalytic subunit beta
PTH: Parathyroid hormone
QTP: Qinghai-Tibetan Plateau
SH3BP2: SH3 domain-binding protein 2
SMPX: Small muscular protein
SSR: Microsatellite
