# Supplementary material for: Genome-wide identification of SNPs associated with body weight in yak
Source: BMC Genomics. 2022 Dec 15;23:833. doi: 10.1186/s12864-022-09077-4 (PMC9756674; doi:10.1186/s12864-022-09077-4)
Supplement: Supplementary file 2 — Additional file 2. [file 12864_2022_9077_MOESM2_ESM.docx]

**Specification of the Affymetrix Yak 600K SNP Array Chip**

The Yak 600K SNP array chip is the first yak high-density SNP chip developed from the genome data of 30 yak breeds from Tibet, Qinghai, Sichuan, Gansu, Yunnan and Xinjiang provinces, covering the main yak breeds in China. It contains 630,209 SNP loci and can be used for whole genome SNP typing of yaks.

The Yak 600K SNP Array Chip is developed as the following process. To generate candidate SNPs for inclusion on an Affymetrix Axiom myDesign Custom Array, we selected 90 representative yaks for genome re-sequencing, which covered 30 yak breeds (3 individuals per breed) from Tibet, Qinghai, Sichuan, Gansu, Yunnan and Xinjiang Provinces. Re-sequencing of these 90 individuals generated a total of 11,409 million paired-end reads, including 1,721.3 Gb of data. The sequencing depth of 36 individuals from 12 recognized domestic yak breeds was approximately 10X, with approximately 5X for the others. The short reads were mapped to the reference genome (BroGru_v2.0) published in 2012, with the mapping coverage rate of 76.29%. The detailed information for the genome re-sequencing could be obtained from Chai et al. (2020).

SNP identification was performed using the GATK software on the data of all individuals. The criteria used for calling SNPs were as following: QD < 2.0, FS > 60, MQ < 40, SOR > 3.0, MQRankSum < -12.5, ReadPosRankSum < -8.0, QUAL < 40, missing rate > 0.5, and minor allele frequency (MAF) ≤ 5%. A total of 12,001,884 SNPs were identified.

For quality control, 71-bp fragments spanning each SNP were extracted, including 35-bp upstream and 35-bp downstream to the SNP base. SNPs with flanking sequences that contained over four consecutive ‘G’ or ‘C’, or over six consecutive ‘A’ or ‘T’, or ‘N’, were removed. Next, GC content was calculated and SNPs with flanking sequences with GC content below 30% or above 70% were removed. The flanking sequences of the remaining SNPs were mapped to the reference genome, and the SNPs that mapped uniquely to a certain location were kept for further selection. SNPs located very close to each other are less likely to be assayed successfully during genotyping because of interference from neighboring variants. Clustering of SNPs can be a result of the misalignment of reads because of the presence of InDels (insertions or deletions) at the beginning or end of reads. Based on advice from Affymetrix scientists, we removed the closely located SNPs that were within 10 bp from each other, and SNPs containing more than two variants within 35 bp were also removed. Priority was given to SNPs in coding sequences. Based on these criteria, finally, a total of 633,899 SNPs were included for the development of the Yak 600K SNP array chip. The information of each SNP site in the chip is listed in the supplementary Table S1.

**References**

Chai ZX, Xin JW, Zhang CF, Dawayangla, Zhong JC. (2020). Whole-genome resequencing provides insights into the evolution and divergence of the native domestic yaks of the Qinghai-Tibet plateau. BMC Evolutionary Biology, 20, 137.
